# Association between retinol intake and periodontal health in US adults

**DOI:** 10.1186/s12903-023-02761-1

**Published:** 2023-02-01

**Authors:** Shenyue Zhou, Juan Chen, Ruoyan Cao

**Affiliations:** 1 The Third Xiangya Hospital of Central South University, Central South University, Changsha, China; 2 Key Laboratory of Shaanxi Province for Craniofacial Precision Medicine Research, College of Stomatology, Xi’an Jiaotong University, No. 98, Xiwu Road, Xincheng District, Xi’an, 710004 China; 3 Department of Periodontology, College of Stomatology, Xi’an Jiaotong University, No. 98, Xiwu Road, Xincheng District, Xi’an, 710004 China; 4 Xiangya Stomatological Hospital & School of Stomatology, Central South University, Changsha, China

**Keywords:** Periodontitis, Retinol, Antioxidant, NHANES

## Abstract

**Background:**

Inflammation and oxidative stress are two hallmarks of periodontitis. Retinol is an antioxidant and suppresses expression of pro-inflammatory factors. However, the evidence for an association between retinol intake and periodontitis is limited. Thus, the aim of this study is to assess the association between retinol intake and periodontal health.

**Methods:**

Data used in this cross-sectional study from the National Health and Nutrition Examination Survey (NHANES) 2009–2014 (n = 9081). Dietary intake of retinol was measured based on two 24-h dietary recall interviews. The category of periodontitis was defined by the CDC/AAP according to clinical periodontal parameters. Univariate and multivariate logistic regression analyses were applied to investigate the relationship between retinol intake and the risk of periodontitis.

**Results:**

Compared with the lowest tertile, individuals in the highest tertile of retinol intake were less likely to be periodontitis (OR_tertile3vs1_ = 0.79, 95% CI: 0.65–0.96). The association was still significant in populations who were less than 60 years old (OR_tertile3vs1_ = 0.80, 95% CI: 0.65–0.97), non-Hispanic black (OR_tertile3vs1_ = 0.62, 95% CI: 0.42–0.94), PI ≤ 1.3 (OR_tertile3vs1_ = 0.72, 95% CI: 0.55–0.93), 1.3 < PI ≤ 3.5 (OR_tertile3vs1_ = 0.70, 95% CI: 0.55–0.89), non-smoker (OR_tertile3vs1_ = 0.63, 95% CI: 0.48–0.81), obesity (OR_tertile3vs1_ = 0.68, 95% CI: 0.49–0.94) and who had not diabetes mellitus (OR_tertile3vs1_ = 0.79, 95% CI: 0.65–0.95) or had hypertension (OR_tertile3vs1_ = 0.63, 95% CI: 0.47–0.84).

**Conclusion:**

Retinol intake is inversely associated with poor periodontal health in US adults.

## Background

Periodontitis is a common chronic inflammatory disease caused by oral biofilm infection, which leads to the progressive destruction of surrounding periodontal tissue [1]. Recent epidemiological evidence indicates that 35% of the general US population suffers from periodontitis, of which approximately 10% are in severe stages [2]. In addition, periodontitis increases the risk of multiple systemic diseases, such as cardiovascular disease and diabetes mellitus [3]. Although there are a growing number of influencing factors for periodontitis, such as flap design [4], few of them can be changed. Thus, it is necessary to identify modifiable influencing factors to improve understanding of periodontitis pathology and facilitate more cost-effective public health efforts targeting periodontitis [5].

Nutrition is an important modifiable factor for periodontitis. For example, pro-inflammatory diets increase the risk of periodontitis, indicating that modulating dietary inflammatory potential may be a useful strategy for the prevention and treatment of periodontal disease [6]. In addition to inflammation, oxidative stress induces proinflammatory mechanisms and osteoclastogenesis, leading to alveolar bone resorption in periodontitis patients [7]. Thus, nutrition with anti-inflammatory and antioxidant capabilities may reduce the risk of periodontitis. Retinol, the active form of vitamin A, is an antioxidant that could scavenge free radicals, inhibit peroxidation and sustain the homeostasis between oxidants and antioxidants [8]. Retinol also has anti-inflammatory ability. For example, it impedes the expression of various proinflammatory cytokines, such as TNF-α, IL-6, IL-12, etc. [9]. Furthermore, retinol has the potential to involve in periodontal reparative. In detail, retinol affects the proliferation and differentiation of the gingival mesenchymal stem [10]. Although the role of retinol has been explored in vitro, the association between retinol intake and periodontists is poorly understood in cross-section study.

Thus, this study aimed to assess the relationship between retinol intake and periodontitis after adjusting for potential confounders based on data from National Health and Nutrition Examination Survey (NHANES). We also explored this association in different subgroups.

## Methods

### Study design and population

The data used in this cross-sectional study were gathered from the NHANES; 2009–2014. Representative population was obtained based on a cluster, stratified and multistage sampling design. Participants with complete full-mouth periodontal examination (FMPE) and complete 24-h recall data were included in this study. Participants younger than 30 years were excluded from this study. Finally, a total of 9081 individuals were included in our study. This study was conducted according to the guidelines laid down in the Declaration of Helsinki, and all procedures involving human subjects were approved by the Research Ethics Review Board of National Center for Health Statistics, and all participants signed informed consent.

### Exposure variable

Dietary intake of retinol was extracted from the total nutrient intake file, including summed nutrients from foods/beverages. All the individuals had two 24 h dietary recalls, and the average intake from these two recalls would be applied in our analysis.

### Outcome variable

The primary outcome of our study was moderate or severe periodontitis. Periodontal examination included probing depth (PD) and clinical attachment loss (CAL) at six sites per tooth without third molars based on the FMPE protocol. A maximum of 168 sites and 28 teeth per subject could be examined to evaluate periodontal status. The category of periodontitis was based on the CDC/AAP definitions [11]. No/mild periodontitis was characterized as no evidence of moderate/severe periodontitis; moderate periodontitis: ≥ 2 interproximal sites with PD ≥ 5 mm not on the same tooth, or ≥ 2 interproximal sites with CAL ≥ 4 mm not on the same tooth; severe periodontitis: ≥ 2 interproximal sites with CAL ≥ 6 mm not on the same tooth and ≥ 1 interproximal sites with PD ≥ 5 mm.

### Potential confounders

Potential confounders were collected from previous studies, including age, gender, race, marital status, education level (< high school, high school, > high school), poverty index (PI; ≤ 1.3, 1.3–3.5, and > 3.5), smoking status (never, former and current) and alcohol consumption (mild, moderate and heavy), obesity (non-obese: BMI < 30 and obese: BMI ≥ 30), total energy intake, diabetes and hypertension.

### Statistical analysis

We considered weights in our analysis according to the NHANES analysis guide (https://wwwn.cdc.gov/nchs/nhanes/tutorials/module3.aspx). The new 6-year weights were calculated using 1/3 of the 2-year dietary weights. Baseline descriptive statistics for this study by tertiles of retinol intake were calculated. Continuous variables were compared based on one-way ANOVA test for normally distributed variables, and Kruskal–Wallis test for non-normally distributed variables. Chi-square test was applied to compare categorical variables.

 Three multivariable logistic models were used to assess the association between retinol intake and moderate/ severe periodontitis after adjusting for potential confounders. Model I was adjusted for age, gender and race, model II was additionally adjusted for education level, marital status, PI, obesity, smoking status, alcohol consumption, diabetes and hypertension, and model III was additionally adjusted for total energy intake. In addition, stratified analyses were conducted based on all variables in Table 1 to assess the association between retinol intake and periodontitis. All the analyses were performed using R software (version 4.1.2). A *P* value less than 0.05 was considered statistically significant.

**Table 1:** Weighted characteristics of the participates

Characteristics	Retinol intake				*P* value
	Overall n = 9081	Tertile 1 n = 3027	Tertile 2 n = 3027	Tertile 3 n = 3027	
Age (year)					0.0007
Mean	50.78	49.96	51.10	51.16	
SD	13.49	12.52	13.48	14.20	
Age group (%)					< 0.0001
≤ 60 years	75.84	79.94	74.37	73.87	
> 60 years	24.16	20.06	25.63	26.13	
Gender (%)					< 0.0001
Female	51.72	58.52	55.22	43.23	
Male	48.28	41.48	44.78	56.77	
Race (%)					< 0.0001
Non-Hispanic White	68.78	59.16	67.26	77.78	
Mexican American	8.02	9.36	8.56	6.47	
Non-Hispanic Black	10.67	14.88	10.8	7.2	
Others	12.53	16.61	13.39	8.54	
Education level (%)					< 0.0001
< High school	14.17	18.55	13.9	10.93	
High school	20.65	21.55	21.4	19.29	
> High school	65.08	59.8	64.52	69.78	
PI (%)					< 0.0001
≤ 1.3	17.8	21.29	17.91	14.93	
1.3–3.5	31.69	34.49	30.13	30.84	
> 3.5	44.47	38.27	45.29	48.68	
Marital status (%)					0.0303
Married/living as married	69.55	67.66	79.6	70.14	
Never married	10.39	9.98	10.4	10.7	
Separated/divorced/widowed	20.03	22.36	18.95	19.12	
Smoking habit (%)					0.0011
Non smoker	56.89	56.06	56.84	57.6	
Former smoker	26.36	24.66	26.83	27.29	
Current smoker	16.73	19.24	16.32	15.11	
Alcohol consumption (%)					< 0.0001
Mild	38.1	33.85	38.58	41.05	
Moderate	18.67	20.17	17.53	18.47	
Heavy	14.58	16.77	14.99	12.48	
Obesity (%)					0.4949
No	62.21	61.68	61.6	63.16	
Yes	37.39	37.8	38.01	36.53	
Diabetes mellitus (%)					0.3126
No	89.74	89.75	89.46	89.97	
Yes	9.48	9.73	9.54	9.22	
Hypertension (%)					< 0.0001
No	60.05	60.18	56.69	62.9	
Yes	39.95	39.82	43.31	37.1	
Total energy intake (Kcal/d)					< 0.0001
Mean	2103.14	1713.15	2022.56	2484.32	
SD	787.62	640.03	652.73	829.01	
Periodontitis (%)					< 0.0001
Non/mild periodontitis	62.78	59.06	63.99	64.69	
Moderate/severe periodontitis	37.22	40.94	36.01	35.31	

## Results

### Characteristics

The 9081 NHANES participants in our study represented 148.6 million noninstitutionalized residents of the United States. Table 1 shows the baseline characteristics from NHANES (2009–2014). On average, individuals were 50.78 years old, with 51.72% females. The average of total energy intake was 2103.14. The percentage of participants with moderate/severe periodontitis was 37.22%. The distribution of diabetes mellitus and obesity were similar among the three groups of retinol intake. Compared with those in the first tertile of retinol intake, individuals in the third tertile tended to be female, non-Hispanic white, more educated, current smokers, less alcohol consumption, non-hypertension and non/mild periodontitis.

### Retinol intake and periodontitis

The weighted of OR (95% CI) of periodontitis based on the tertile of retinol intake were presented in Table 2. High retinol intake was negatively associated with moderate/severe periodontitis in the crude model and adjusted models: Crude model (OR_tertile2vs1_ = 0.81, 95% CI: 0.68–0.96; OR_tertile3vs1_ = 0.79, 95% CI: 0.68–0.92), Model I (OR_tertile2vs1_ = 0.76, 95% CI: 0.65–0.90; OR_tertile3vs1_ = 0.73, 95% CI: 0.61–0.86), Model II (OR_tertile2vs1_ = 0.82, 95% CI: 0.68–0.98; OR_tertile3vs1_ = 0.82, 95% CI: 0.68–0.98), and Model III (OR_tertile2vs1_ = 0.80, 95% CI: 0.67–0.96; OR_tertile3vs1_ = 0.79, 95% CI: 0.65–0.96).

**Table 2:** Association between retinol intake and periodontitis

	Retinol intake					*P* Trend
	Tertile 1	Tertile 2	*P* value	Tertile 3	*P* value	
Crude model	1	0.81 (0.68, 0.96)	0.021	0.79 (0.68, 0.92)	0.004	0.004
Model I	1	0.76 (0.65, 0.90)	0.002	0.73 (0.61, 0.86)	0.0007	0.0008
Model II	1	0.82 (0.68, 0.98)	0.038	0.82 (0.68, 0.98)	0.046	0.051
Model III	1	0.80 (0.67, 0.96)	0.028	0.79 (0.65, 0.96)	0.027	0.029

Model I: Adjusted for age, gender and race

Model II: Model I and adjusted for education level, marital status, PI, obesity, smoking status, alcohol consumption, diabetes mellitus and hypertension

Model III: Model II and adjusted for total energy intake

### Subgroup analyses

The subgroup analyses on the association of retinol intake with moderate/severe periodontitis were shown in Table 3. High retinol intake was negatively associated with moderate/severe periodontitis in those who were less than 60 years old (OR_tertile2vs1_ = 0.78, 95% CI: 0.63–0.96; OR_tertile3vs1_ = 0.80, 95% CI: 0.65–0.97), non-Hispanic black (OR_tertile2vs1_ = 0.71, 95% CI: 0.51–0.98; OR_tertile3vs1_ = 0.62, 95% CI: 0.42–0.94), PI ≤ 1.3 (OR_tertile2vs1_ = 0.68, 95% CI: 0.51–0.92; OR_tertile3vs1_ = 0.72, 95% CI: 0.55–0.93), 1.3 < PI ≤ 3.5 (OR_tertile2vs1_ = 0.69, 95% CI: 0.52–0.93; OR_tertile3vs1_ = 0.70, 95% CI: 0.55–0.89), non-smoker (OR_tertile2vs1_ = 0.67, 95% CI: 0.52–0.86; OR_tertile3vs1_ = 0.63, 95% CI: 0.48–0.81), obesity (OR_tertile2vs1_ = 0.72, 95% CI: 0.55–0.95; OR_tertile3vs1_ = 0.68, 95% CI: 0.49–0.94), who had not diabetes mellitus (OR_tertile3vs1_ = 0.79, 95% CI: 0.65–0.95) or who had hypertension (OR_tertile3vs1_ = 0.63, 95% CI: 0.47–0.84).

**Table 3:** Weighted OR (95% CI) of retinol intake and periodontitis in each subgroup

Subgroup	Retinol intake				
	Tertile 1	Tertile 2	*P* value	Tertile 3	*P* value
Age					
≤ 60	1	**0.78 (0.63, 0.96)**	0.027	**0.80 (0.65, 0.97)**	0.036
> 60	1	0.83 (0.61, 1.14)	0.264	0.77(0.55, 1.07)	0.131
Gender					
Female	1	0.86 (0.70, 1.07)	0.194	0.76 (0.59, 1.00)	0.063
Male	1	**0.72 (0.53, 0.98)**	0.046	0.77 (0.56, 1.06)	0.121
Race					
Non-Hispanic White	1	0.87 (0.66, 1.15)	0.335	0.85 (0.65, 1.12)	0.258
Mexican American	1	0.76 (0.54, 1.06)	0.128	0.89 (0.58, 1.37)	0.614
Non-Hispanic Black	1	**0.71 (0.51, 0.98)**	0.049	**0.62 (0.42, 0.94)**	0.034
Others	1	0.80 (0.54, 1.21)	0.301	0.73 (0.51, 1.03)	0.090
Education level					
< High school	1	0.80 (0.58, 1.10)	0.183	0.66 (0.43, 1.01)	0.070
High school	1	0.82 (0.56, 1.18)	0.295	0.88 (0.61, 1.27)	0.518
> High school	1	0.81 (0.63, 1.06)	0.134	0.81 (0.64, 1.03)	0.093
PI					
≤ 1.3	1	**0.68 (0.51, 0.92)**	0.021	**0.72 (0.55, 0.93)**	0.020
1.3–3.5	1	**0.69 (0.52, 0.93)**	0.021	**0.70 (0.55, 0.89)**	0.008
> 3.5	1	0.93 (0.65, 1.34)	0.712	0.89 (0.62, 1.26)	0.505
Marital status					
Married/living as married	1	0.85 (0.68, 1.07)	0.172	0.79 (0.61, 1.01)	0.077
Never married	1	0.72 (0.44, 1.19)	0.218	1.05 (0.60, 1.83)	0.869
Separated/divorced/widowed	1	**0.69 (0.50, 0.95)**	0.033	0.73 (0.52, 1.03)	0.089
Smoking habit					
Non smoker	1	**0.67 (0.52, 0.86)**	0.004	**0.63 (0.48, 0.81)**	0.002
Former smoker	1	1.05 (0.73, 1.50)	0.800	1.06 (0.74, 1.50)	0.769
Current smoker	1	0.89 (0.62, 1.28)	0.534	1.00 (0.68, 1.46)	0.982
Alcohol consumption					
None/Mild	1	1.11 (0.81, 1.52)	0.540	0.87 (0.62, 1.21)	0.418
Moderate	1	**0.69 (0.50, 0.97)**	0.041	0.73 (0.50, 1.06)	0.107
Heavy	1	0.85 (0.52, 1.37)	0.507	0.79 (0.51, 1.21)	0.289
Obesity					
No	1	0.88 (0.70, 1.12)	0.316	0.86 (0.68, 1.09)	0.235
Yes	1	**0.72 (0.55, 0.95)**	0.027	**0.68 (0.49, 0.94)**	0.029
Diabetes mellitus					
No	1	0.81 (0.66, 0.99)	0.057	**0.79 (0.65, 0.95)**	0.018
Yes	1	0.74 (0.44, 1.23)	0.251	0.80 (0.43, 1.52)	0.507
Hypertension					
No	1	0.84 (0.65, 1.09)	0.200	0.91 (0.72, 1.15)	0.455
Yes	1	0.73 (0.54, 1.00)	0.065	**0.63 (0.47, 0.86)**	0.009

Bold denotes statistical signifcance at *P* < 0.05

Adjusted for age, gender, race, education level, marital status, PI, obesity, smoking status, alcohol consumption, diabetes mellitus, hypertension and total energy intake except the subgroup variable

## Discussion

In this cross-sectional study, we found that retinol intake was inversely associated with moderate/severe periodontitis in the US adult population. In addition, such association remained significant in populations who were less than 60 years old, non-Hispanic black, PI ≤ 1.3, 1.3 < PI ≤ 3.5, non-smoker, obesity and who had not diabetes mellitus or had hypertension. Our study offers a potential new strategy for the prevention and treatment of periodontal disease.

Oxidative stress is a hallmark of multiple diseases, including periodontitis. Recent study indicates that human gingival fibroblasts from periodontitis exhibits enhanced ROS production [12]. ROS has been regarded as a “double-edged” sword in periodontitis. ROS is produced by neutrophils to eliminate invading pathogenic microorganisms in healthy periodontal tissue, however, excessive ROS can trigger cytotoxic to host cells by damaging DNA, lipids and proteins [13]. Thus, it is necessary to reduce ROS production to improve the prognosis of periodontitis. Antioxidant compounds, such as resveratrol and curcumin, inhibit osteoclastogenesis and reduce alveolar bone loss, and thus they have the potential to provide additional benefits for scaling and root planning [7]. In addition, dietary or nutritional interventions show favorable effects on periodontal treatment outcomes and prevention of periodontitis [14]. For example, supplementation of vitamin C can decrease the risk of periodontitis and suppress the senescence of periodontal ligament stem cells [15–17]. However, the association between periodontitis and intake of another antioxidant, retinol, is unclear.

Retinol, the active form of vitamin A, must be obtained from the diet and is involved in diverse biological functions, including proliferation, apoptosis, differentiation and metabolism [18]. Mammalian cells uptake retinol via STRA6 and retinol can be metabolized to retinal and then retinoic acid [19]. Retinoic acid exhibits inhibition of periodontal inflammation. In detail, an oral supplement of all-trans retinoic acid in periodontitis mouse model suppresses inflammatory cell infiltration and Th17 cell activation and enhances Treg cell activation, thereby reducing alveolar bone loss [20].

In addition, retinol itself might hamper progression of periodontitis. It reduces the expression of serval inflammatory factors such as TNF-β, IL-6, IL-1α and IL-1β [21, 22]. Most importantly, retinol has the ability to drive cellular de-differentiation and pluripotency [23], which is vital to periodontal tissue regeneration. Thus, it is reasonable to find that higher retinol intake reduces the risk of periodontitis.

In line with our results, in young Korean women, a marginal association between lower retinol intake and periodontitis is found (OR = 1.56, 95% CI: 1.00–2.44) [24]. However, such association is not observed in men aged 60–70 from Northern Ireland [25]. In our study, we also did not find association between retinol intake and periodontitis in population over the age of 60. This might be because aging is a risk factor of periodontitis, increasing prevalence and progression of periodontitis [26]. Thus, it is still necessary to seek other modifiable influencing factors for elderly patients with periodontitis.

In subgroup analysis, we found that retinol intake was inversely associated with moderate/severe periodontitis in serval groups, such as non-Hispanic black, non-smoker and obesity. Bacterial composition in periodontal tissue might partly account for this result. Metagenomics indicates that smoking, geographical location and ethnicity play an important role in bacterial composition. For example, oral biofilm in smokers consists of more periodontopathogen bacteria compared to non-smokers [27].

It has demonstrated that periodontitis was associated with multiple systemic diseases, including cardiovascular disease. Anticoagulant drugs are common for cardiovascular disease and thus increase the risk of periodontal treatment in these patients [28]. Previous studies indicate that all-trans retinoic acid could ameliorate the coagulopathy. In detail, all-trans retinoic acid reduces plasma levels of fibrinopeptide A, prothrombin fragment 1.2, thrombin antithrombin complex and fibrin d-dimer [29]. Thus, retinol intake may not be appropriate for patients with cardiovascular disease who are about to undergo periodontal treatment.

Our study has several limitations. First, it is unfeasible to identify causal relationships between retinol intake and periodontitis based on cross-sectional designs. Future multi-center prospective trails are required to corroborate the potential causal association and help confirm the benefits of increasing retinol intake on periodontitis health. Second, two 24-h diet recall interviews may not be ideal for reflecting long-term dietary intake. Finally, we could not rule out all possible residual confounders due to unmeasured confounding factors.

## Conclusion

Retinol intake is inversely associated with periodontitis among US adults. Further prospective trials are required to support our findings.

## Data Availability

The NHANES data of our study are openly available at https://www.cdc.gov/nchs/nhanes/default.aspx.

## Abbreviations

NHANES: National Health and Nutrition Examination Survey
ROS: Reactive oxygen species
FMPE: Full-mouth periodontal examination
PD: Probing depth
CAL: Clinical attachment loss
